# The value of vector ECG in predicting residual pulmonary hypertension in CTEPH patients after pulmonary endarterectomy

**DOI:** 10.1371/journal.pone.0317826

**Published:** 2025-02-26

**Authors:** Dieuwke Luijten, Tamara Rodenburg, Harm-Jan Bogaard, Azar Kianzad, Dieuwertje Ruigrok, Philip Croon, Patrick Smeele, Hubert W. Vliegen, Anton Vonk Noordegraaf, Lilian J. Meijboom, Frederikus A. Klok

**Affiliations:** 1 Department of Thrombosis and Hemostasis, Leiden University Medical Center, Leiden, the Netherlands; 2 Department of Pulmonary Medicine, Amsterdam University Medical Centers, Amsterdam, the Netherlands; 3 Amsterdam Cardiovascular Sciences, Pulmonary Hypertension and Thrombosis, Amsterdam, the Netherlands; 4 Department of Cardiology, Amsterdam University Medical Centers, Amsterdam, the Netherlands; 5 Department of Cardiology, Leiden University Medical Center, Leiden, the Netherlands; 6 Department of Radiology and Nuclear Medicine, Amsterdam University Medical Centers, Amsterdam, the Netherlands; University of Colorado - Anschutz Medical Campus, UNITED STATES OF AMERICA

## Abstract

**Introduction:**

Right heart catheterization (RHC) is the diagnostic standard for establishing residual pulmonary hypertension (PH) after pulmonary endarterectomy (PEA) in patients with chronic thromboembolic pulmonary hypertension (CTEPH). A potential non-invasive alternative diagnostic test could be electrocardiography (ECG)-derived ventricular gradient optimized for right ventricular pressure overload (VG-RVPO).

**Methods:**

We studied 66 CTEPH patients who underwent PEA. A subgroup of 20 patients also had a cardiac MRI before and after PEA. The diagnostic performance of the VG-RVPO for the detection of residual PH as well as the potential to replace RHC were assessed. Different cut-off values to define a normal VG-RVPO were evaluated. Also, we evaluated the association between mean pulmonary artery pressure (mPAP) and CMR derived indexed right ventricular (RV) mass and the VG-RVPO.

**Results:**

During follow-up, 28 patients had residual PH (42%). A decrease in VG-RVPO after PEA was associated with decrease in mPAP or indexed RV mass post PEA (r = 0.55, p < 0.05 and r = 0.64, p < 0.05, respectively). If a normal VG-RVPO would exclude residual PH, the need for RHC would be reduced with 15–48%, but up to 36% of the CTEPH patients with residual PH would have been missed as they had a normal VG-RVPO.

**Conclusion:**

Although there was an association between the change in VG-RPVO and changes in mPAP or indexed RV mass, our study demonstrated that VG-RPVO has limited value in excluding the presence of residual PH post-PEA as up to 36% of the CTEPH patients with residual PH would have been missed if residual PH would have been excluded based on a normal VG-RVPO.

## Introduction

Pulmonary endarterectomy (PEA) is the treatment of choice for patients with chronic thromboembolic pulmonary hypertension (CTEPH) [1–3]. PEA leads to improved cardiopulmonary hemodynamics and exercise tolerance with low early mortality when performed in expert centres [1,4,5]. Nevertheless, residual pulmonary hypertension (PH) after PEA is not uncommon, and associated with worse long-term survival [6,7]. For patients with significant residual PH after PEA, balloon pulmonary angioplasty (BPA) or PAH (pulmonary arterial hypertension)-specific medication are potential treatment options to lower symptom burden.

Right heart catheterization (RHC) is the diagnostic standard for diagnosing post-PEA residual PH. Current guidelines therefore advice to perform RHC 3–6 months after surgery. However, a non-invasive strategy to perform post-PEA follow-up might be preferred. A potential non-invasive alternative is the ECG-derived ventricular gradient optimized for right ventricular pressure overload (VG-RVPO) [8–10]. The VG-RVPO detects right ventricle pressure overload due to right ventricle hypertrophy and changes in action potential duration as a result from pressure variations [10]. In a normal heart the ventricular gradient points in a left direction, therefore a normal VG-RVPO is negative. With increase of right ventricle (RV) pressure, the VG-RVPO becomes more positive and can therefore detect RV pressure overload [8].

Given that the VG-RVPO generates numerical values, it can be categorized into absence or presence of signs of right ventricle pressure overload using previous derived cut-off values [11–14]. The diagnostic value of VG-RVPO for post-PEA residual PH has not been established to date. Therefore, our aim was to evaluate the diagnostic accuracy of the ECG-derived VG-RVPO for detecting residual PH in CTEPH patients who underwent PEA. To our knowledge this is the first study to investigate the diagnostic accuracy of vector ECG in detecting residual PH in CTEPH patients who underwent PEA.

## Methods

### Study design and patients

This was a post-hoc analysis of the VUmc observational CTEPH follow-up cohort (Amsterdam, the Netherlands) [15]. All CTEPH patients undergoing PEA between July 2012 and September 2019 were eligible for inclusion. Patients were excluded if 1) they had a follow-up of < 6 months after PEA; 2) they did not have an available baseline ECG (i.e., ECG within one month before CTEPH diagnosis or between CTEPH diagnosis and PEA); or 3) they did not have a follow-up ECG (i.e., ECG 6-21 months after PEA). Of the included patients deidentified data from the patient chart was saved in a database. Patients were diagnosed with CTEPH according to the at inclusion applicable guideline definition (mPAP ≥ 25 mmHg) [16]. Per clinical protocol, ECG and cardiac MRI (CMR) was routinely performed before and 6 months after PEA. Residual PH was defined as mPAP ≥ 25 mmHg measured with RHC following at the inclusion applicable guideline definitions for pulmonary hypertension. The study did not fall within the scope of the Medical Research Involving Human Subjects Act, because an analysis was performed based on available clinical data obtained for clinical purposes and therefore no informed consent was obtained. This was confirmed by the Medical Ethics Review Committee of the VU University Medical Center (2017.313).

### Objectives

The primary objective of this study was to investigate the diagnostic accuracy of the (Δ or follow-up) VG-RVPO to detect residual PH in CTEPH patient who underwent PEA, and its efficacy and safety for making management decisions. For efficacy we evaluated the percentage of patients in whom residual PH could not have been ruled out with the VG-RVPO, i.e., the number of patients who would have had an RHC indication. For safety we evaluated the percentage of patients in whom residual PH would have been missed if residual PH would have been ruled out based on a normal VG-RVPO.

Secondary objectives were (1) to investigate the optimal cut-off value of the (Δ or follow-up) VG-RVPO for the detection of residual PH and the subsequent diagnostic accuracy, efficacy and safety of this cut-off value, (2) to evaluate the correlation between VG-RVPO and the mean pulmonary artery pressure (mPAP) as measured by right heart catheterization (RHC), (3) to evaluate the correlation between VG-RVPO and right ventricular (RV) hypertrophy as measured by indexed RV mass on CMR and (4) to evaluate the diagnostic accuracy of the VG-RVPO in patients with normal versus abnormal indexed RV mass on CMR.

### Procedures

**Fig 1:**
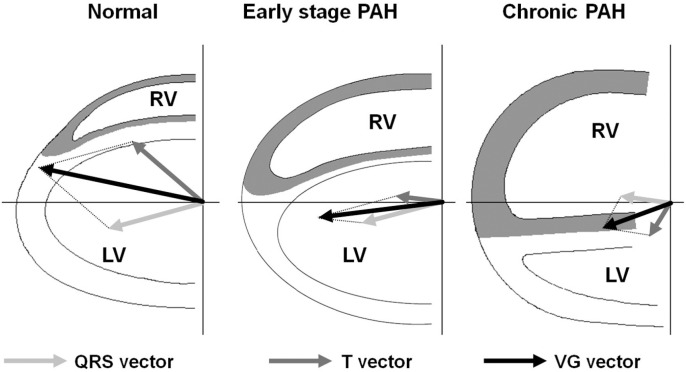
Change in cardiac vectors from the normal physiologic situation to respectively early stage and chronic PH. Reprinted from Couperus et al. with permission [13]. Pulmonary arterial hypertension PAH.

RHC was performed as described previously [17]. ECGs were standard 10-s 12 lead ECGs recorded in supine position (25 mm/s). To determine the ECG variables, the dedicated Leiden ECG analysis and decomposition software program (LEADS) was performed by an independent investigator blinded to patient characteristics and outcomes [18]. The LEADS software computes multiple vector-cardiogram (VCG) values including the ventricular gradient (VG). The VG is defined as the 3D integral of the heart vector over the QT interval and is an indicator for how the action potential morphology is distributed in the heart [19]. For the detection of right ventricular pressure overload (RVPO) previous research has shown that the projection in the 155 ° azimuth and 27 ° elevation direction is the most optimal, since this projection is directed over the right ventricle [8,9,11–13]. This projection is called the VG-RVPO (ventricular gradient – optimized for right ventricular pressure overload). Since in a normal heart the VG points in a left direction, a normal VG-RVPO is negative and with increase of right ventricular pressure the VG-RVPO becomes more positive (**Fig 1**). The VG-RVPO cut-off point for the detection of pulmonary hypertension derived from previous studies is < −13 mV ms; meaning that a VG-RVPO < −13 mV · ms was considered normal (no residual PH) and a VG-RVPO of ≥  −13 mV · ms was considered abnormal (possible residual PH) although different cut-off points have been evaluated in this study [11–14]. Baseline VG-RVPO was derived from the last ECG performed before PEA, follow-up VG-RVPO was derived from the ECG performed approximately 6 months after PEA. Δ VG-RVPO was calculated as follows: follow-up VG-RVPO - baseline VG-RVPO.

CMR were performed on a 1.5 T Sonata or 1.5 T Avanto MRI scanner (Siemens Healthcare, Erlangen, Germany). A short-axis stack was performed at breath-hold per slice, with a slice thickness and interslice gap of 5 mm. RV volume and mass were determined by manually drawing endocardial and epicardial contours at end diastole and end systole using commercially available software (QMass, Medis, Leiden, the Netherlands and Circle CVI42). RV mass was subsequently indexed to body surface area [20]. As healthy controls have an indexed RV mass of 22 ± 6 g/m^2^ we defined an abnormal indexed RV mass as > 33.76 g/m^2^ which is the upper limit of the 95% CI in healthy controls [20].

### Statistical analysis

Normally distributed continuous data were described as a mean (±standard deviation [SD]). Abnormally distributed continuous data were described as a median (interquartile range [IQR]) and compared using a Mann-Whitney-U test. Categorical variables were described as numbers (percentage).

For the analysis of diagnostic accuracy of the VG-RVPO for post-PEA residual PH, sensitivity and specificity of the VG-RVPO (according to the predefined cut-off of ≥  −13 mV·ms) with corresponding confidence interval (95%CI) were calculated. Moreover, ROC curves were plotted, and the area under the curve (AUC) with corresponding 95%CI was determined.

We subsequently calculated the optimal cut-off points for VG-RVPO after PEA and for ΔVG-RVPO by selecting cut-off values to define abnormality according to the highest negative predictive value. For these newly selected cut-off point, we also calculated the diagnostic accuracy as described above.

To evaluate the correlation between the VG-RVPO and mPAP and VG-RVPO and indexed RV mass, scatter plots were drawn and a Pearson correlation coefficient was calculated to quantify the strength of the using linear regression analysis.

**Fig 2:**
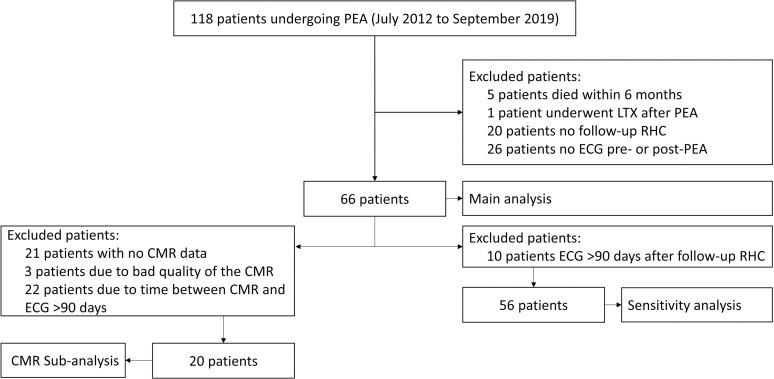
Study flow chart. Abbreviations: CMR, cardiac magnetic resonance imaging; ECG, electrocardiogram; LTX, lung transplantation; PEA, pulmonary endarterectomy; RHC, right heart catheterization. *  11 patients with no CMR post PEA.

Also we stratified all diagnostic accuracy outcomes according to normal or abnormal RV mass. Patients with bad quality CMR or those with more than 90 days between the CMR and ECG were excluded from this sub-analysis (Fig 2).

We performed two sensitivity analyses: 1) residual PH defined according to the 2022 pulmonary hypertension guidelines from European Society of Cardiology (ESC); pulmonary artery pressure >  20 mmHg, pulmonary artery wedge pressure < 15 mmHg and a pulmonary vascular resistance > 160 dynes^.^s^.^cm^−5^, and 2) excluding all patients where follow-up ECG was performed >  90 days after follow-up RHC.

All analyses were performed using R, version 4.3.1 (R Foundation for Statistical Computing; www.R-project.org).

## Results

### Patients

Sixty-six CTEPH patients who underwent PEA and survived a minimum of 6 months were studied (**Fig 2**). Mean age was 57 years and 56% was male (**Table 1**); 86% had a history of acute pulmonary embolism and 35% of deep vein thrombosis. Before PEA, most patients had a New York Heart Association (NYHA) score of II and 37% used PH-specific medication. Pre-PEA RHC showed a mean mPAP of 42.5 mmHg (interquartile range [IQR]: 35–50) and a mean PVR of 600 dynes^.^s^.^cm^−5^ (IQR 376-748). During follow-up, 28 patients were found to have residual PH (42%) with a mean mPAP of 31.0 mmHg and PVR of 303 dynes^.^s^.^cm^−5^ and 38 patients were found to have no residual PH with a mean mPAP of 19.2 mmHg and PVR of 176 dynes^.^s^.^cm^−5^. When using the new criteria to define pulmonary hypertension based on the 2022 ESC guideline 30 patients were found to have residual PH (46%) with a mean mPAP of 28.2 mmHg and PVR of 295 dynes^.^s^.^cm^−5^ and 35 (54%) patients were found to have no residual PH with a mean mPAP of 20.9 mmHg and PVR of 136 dynes^.^s^.^cm^−5^.

**Table 1:** Baseline characteristics.

	Overall (n = 66)
Age at PEA [years], mean (SD)	57.3 (14.1)
Male sex, n (%)	37 (56.1)
BMI [kg·m − 2], mean (SD)	27.0 (5.9)
NYHA class, n (%)	
I	1 (1.6)
II	24 (38.1)
III	32 (50.8)
IV	6 (9.5)
Use of PH-specific medication before PEA, n (%)	24 (36.9)
Comorbidities, n (%)	
Acute PE	55 (85.9)
DVT	20 (34.5)
History of a malignancy	3 (4.6)
History of a haematological disease	2 (3.1)
Diabetes mellitus	5 (7.7)
Obstructive lung disease	8 (12.3)
Hypertension	22 (33.8)
Splenectomy	1 (1.5)
Coronary artery disease	2 (3.1)
Thyroid disease	5 (7.7)
Months between PEA to follow-up ECG/RHC, median (IQR)	6.93 (6.46–8.23)
Mean mPAP pre PEA [mmHg], mean(SD)	42.5 (10.2)*
Mean PVR pre-PEA [dynes^.^s^.^cm^−5^], mean (SD)	600.7 (299.5)

*patients without residual PH during follow-up had a mPAP pre PEA of 41.95 mmHg (SD 10.69), which was 43.18 mmHg (9.77) for patients with residual PH during follow-up. Abbreviations: BMI, body mass index; DVT, deep vein thrombosis; ECG, electrocardiogram; IQR, interquartile range; mPAP, mean pulmonary artery pressure; NYHA, New York Heart Association; PE, pulmonary embolism; PEA, pulmonary endarterectomy; PH, pulmonary hypertension; PVR, pulmonary vascular resistance; RHC, right heart catheterization; SD, standard deviation.

### Diagnostic accuracy of VG-RVPO

If residual PH would have been considered ruled out based on a normal follow-up VG-RVPO of < −13 mV·ms, specificity and sensitivity for detecting residual PH would have been 50% and 64% respectively. RHC would have been indicated in 37 patients (56%), but residual PH would have been missed in 10 out of 28 patients (35.7%; **Table 2**), with a negative predictive value of 65.6%.

**Table 2:** Diagnostic accuracy of specific cut-off values.

		Patients without residual PH after PEA (n = 38)	Patients with residual PH after PEA (n = 28)
Abnormal follow-up VG-RVPO of ≥ −13 mV·ms (previously defined cut-off value)	VG-RVPO normal, n (%)	19 (50)	10 (35.7)
	VG-RVPO abnormal, n (%)	19 (50)	18 (64.3)
Abnormal follow-up VG-RVPO of ≥ −14.7 mV·ms	VG-RVPO normal, n (%)	18 (47.3)	9 (32.1)
	VG-RVPO abnormal, n (%)	20 (52.6)	19 (67.9)
Abnormal Δ VG-RVPO of ≥ −24.9 mV·ms	VG-RVPO normal, n (%)	8 (21.1)	2 (7.1)
	VG-RVPO abnormal, n (%)	30 (79.0)	26 (92.9)

Abbreviations: PEA, pulmonary endarterectomy; PH, pulmonary hypertension; SD, standard deviation; VG-RVPO, ventricular gradient optimized for right ventricular pressure overload.

Based on the highest negative predictive value, the best cut-off value for a normal follow-up VG-RVPO would be < −14.7 mV·ms (negative predictive value of 66.7%), and the best cut-off a normal ΔVG-RVPO would have been < −24.9 mV·ms (negative predictive value of 80%). For the newly defined cut-off for follow-up VG-RVPO, the specificity would have been 47.3% and sensitivity 67.9%. RHC would have been indicated in 39 patients (59%), but residual PH would have been missed in 9 patients (32.1%). For the newly defined cut-off for ΔVG-RVPO, the specificity would have been 21.1% and sensitivity 92.9%. RHC would have been indicated in 56 patients (84%), but residual PH would have been missed in 2 patients (7.1%).

The overall predictive accuracy of follow-up RVPO and Δ VG-RVPO for detection of CTEPH was moderate to poor, with a AUCs of the ROC ranging from 0.546 to 0.626 (**Table 3**).

**Table 3:** AUC ROC curve.

	AUC (95%CI
follow-up VG-RPVO	0.546 (0.396–0.697)
follow-up VG-RVPO ≥ −13 mV·ms	0.571 (0.45–0.692)
follow-up VG-RVPO ≥ −14.7 mV·ms	0.576 (0.457–0.695)
Δ VG-RVPO	0.626 (0.488–0.764)
Δ VG-RVPO ≥ −24.9 mV·ms	0.570 (0.488–0.695)

Abbreviations: AUC, area under the curve; VG-RVPO, ventricular gradient optimized for right ventricular pressure overload

When evaluating diagnostic accuracy of the ΔVG-RVPO to detect residual PH for patients with normal vs abnormal indexed RV mass, using the VG-RVPO only in patients with a normal indexed RV mass, would not have improved the performance. Specificity would have been 36–55% and sensitivity 57–100(S1 Table, S1 Fig). Only when using the cut-off for Δ VG-RVPO of < −24.9 mV·ms there would have been an indication to perform RHC in 78% of the patients with a normal indexed RV mass and none of the residual PH patients with a normal indexed RV mass would have been missed. However, as the CMR sub analysis could only be performed in 20 patients and the indexed RV mass post-PEA was abnormal in only 2 patients, power was very low and these results are highly uncertain.

### Sensitivity analyses for the diagnostic accuracy of VG-RVPO

When using the new criteria to define pulmonary hypertension based on the 2022 ESC guideline, we saw similar results for the mean VG-RVPO measurement in patients with/without residual PH (S2 Table). For the diagnostic performance of the VG-RVPO using the different cut-off values, specificityranged between 20–51% and sensitivity between 67–93%. Also, the need for RHC was minimized to 57–86%, but residual PH would have been missed in 7–33% of the patients with residual PH (S3 Table). Overall predictive accuracy was moderate to poor (AUC ROC ranged 0.561–0.774; **S4 Table**).

When excluding the 10 patients with an ECG > 90 days after follow-up RHC, mean VG-RVPO and diagnostic accuracy showed similar results as the main analysis (S5– S7 Tables).

### VG-RVPO measurements before and after PEA

At baseline, mean VG-RVPO was −5.14 mV·ms. Post-PEA, this was −11.2 mV·ms (Table 4). There was no clear difference in post-PEA VG-RVPO between patients with and without residual PH (−10.0 vs −12.1 mV·ms, respectively; mean difference 2.07, 95% CI −5.36 to 9.49). Overall, Δ VG-RVPO was −6.07 mV·ms, indicating a more negative VG-RVPO over time (i.e., more ‘normal’). Patients with residual PH had a numerical lower Δ VG-RVPO compared to patients without residual PH (−2.36 vs −8.81 mV·ms, respectively; mean difference 6.46 mV·ms, 95% CI −2.28 to 15.2)

**Table 4:** VG-RVPO measurements.

	All patients (n = 66)	Patients without residual PH after PEA (n = 38)	Patients with residual PH after PEA (n = 38)	Mean difference (95%CI)
VG-RVPO at baseline (mV·ms), mean +- SD	−5.14 (18.2)	−3.28 (18.5)	−7.67 (17.8)	−4.39 (95% CI −13.4–4.62)
VG-RVPO during follow-up (mV·ms), mean +- SD	−11.2 (13.6)	−12.09 (9.55)	−10.0 (17.9)	2.07 (95% CI −5.36–9.49)
Δ VG-RVPO (between baseline and during follow up) (mV·ms), mean +- SD	−6.07 (17.8)	−8.81 (17.9)	−2.36 (17.3)	6.46 (95% CI −2.28–15.2)

Abbreviations: PEA, pulmonary endarterectomy; PH, pulmonary hypertension; SD, standard deviation; VG-RVPO, ventricular gradient optimized for right ventricular pressure overload.

### Association VG-RVPO with mPAP and indexed RV mass

**Fig 3:**
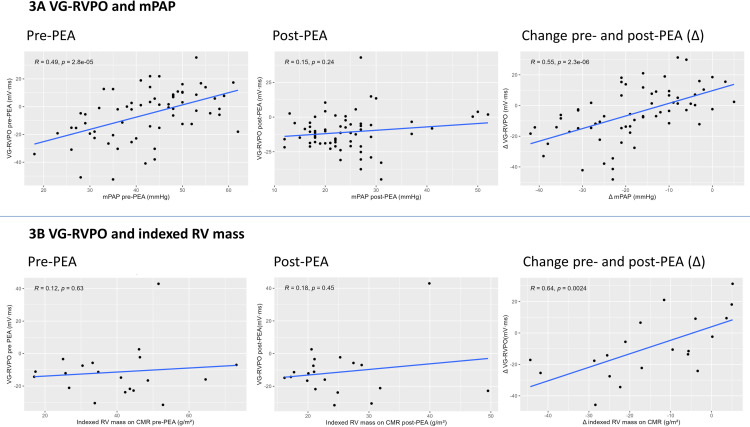
Relationship between VG-RVPO and mPAPor indexed RV mass. Abbreviations: CMR, cardiac magnetic resonance; mPAP, mean pulmonary artery pressure; PEA, pulmonary endarterectomy; RV, right ventricle; VG-RVPO, ventricular gradient optimized for right ventricular pressure overload.

Fig 3a depicts the association between the VG-RVPO and the mPAP measured at RHC. Before PEA, a higher mPAP is correlated with a higher VG-RVPO (r = 0.49, p < 0.05). After PEA this correlation seems to dilute, as the correlation coefficient (r) is only 0.15 (p = 0.24). However, when looking at Δ VG-RVPO and mPAP, a positive correlation was identified (r = 0.55, p < 0.05). Fig 3b depicts the correlation between VG-RVPO and indexed RV mass. There seems to be a positive correlation between VG-RVPO and indexed RV mass before PEA (r = 0.12, p = 0.63), after PEA (r = 0.18, p = 0.45), and over time (Δ; r = 0.64, p < 0.05).

## Discussion

The main goal of this study was to evaluate the diagnostic accuracy of the VG-RVPO for detecting residual PH in CTEPH patients post PEA. Unfortunately, although the pre-PEA and Δ VG-RVPO significantly correlated with mPAP and indexed RV mass, the use of the VG-RVPO in detecting residual PH was limited as 36% of the CTEPH patients with residual PH had a normal VG-RVPO and 7% had a clear improvement of VG-RVPO over time. This suggests that relying solely on VG-RVPO for the detection of residual PH would result in overlooking a substantial portion of affected individuals.

Chronically increased pulmonary artery pressure resulting in RV pressure overload includes changes in action potential duration that can be detected using vector ECG. The VG-RVPO, a vector gradient optimized to detect RV pressure overload, operates on this principle. Given that VG-RVPO measurement is a non-invasive tool, we hypothesized its potential utility in detecting persistent increased pulmonary artery pressure (i.e., residual PH) in CTEPH patients following PEA. Indeed before PEA there was correlation between increased mPAP or indexed RV mass and the VG-RVPO. However, our study found that VG-RVPO did not perform adequately in excluding the presence of residual PH after PEA, likely due to remodeling of the heart after PEA.

One of the factors contributing to the underperformance of the VG-RVPO related to remodeling of the heart after PEA might be persistent RV hypertrophy. Following PEA there is a reduction in RV mass, although it does not fully normalize compared to healthy controls [20]. In some CTEPH patients, RV hypertrophy may persist despite normalization of pulmonary artery pressure post-PEA, leading to an abnormal VG-RVPO. This could diminish the discriminative ability of the VG-RVPO in detecting residual PH. However, even in patients with a normalized RV mass, the diagnostic accuracy of the VG-RVPO for detecting residual PH was poor. Although it should be noted that the power of this analysis was severely limited due to the availability of CMR data in only 20 patients. Therefore, definitive conclusions regarding this sub-analysis cannot be drawn.

Given that the VG-RVPO was designed to detect electrophysiological changes in action potential duration rather than sole RV hypertrophy [8], it’s crucial to consider other factors related to the remodeling of the heart after PEA that may impact its performance in discriminating residual PH. One such factor post-PEA could be the persistent abnormality in the composition of the heart. Despite the decrease in RV mass post-PEA, Braams and colleagues have demonstrated that the composition of the heart after PEA remains abnormal [20]. The persistent altered composition might lead to heterogeneity in action potential duration, thus influencing the ability of the VG-RVPO to detect increased pulmonary artery pressure. Therefore, beyond RV hypertrophy, the ongoing abnormality in the heart’s composition post-PEA could contribute to the suboptimal performance of VG-RVPO in this context.

Overall, the VG-RVPO seems to effectively detect increased pulmonary artery pressure before PEA which aligns with a previous study demonstrating that the VG-RVPO significantly correlated with increased mPAP in patients with suspected PH and effectively identifies PH in systemic sclerosis patients [8,9]. However, due to heart remodeling post-PEA, the additional value of the VG-RVPO in identifying residual pulmonary hypertension (PH) post-PEA is limited. A previous study also showed limited use of the VG-RVPO in the detection of CTEPH in acute PE survivors, possibly due to the diluting effect of persistent RV pressure overload in non-CTEPH acute PE survivors [21]. Whether the VG-RVPO could still contribute to the diagnostic of suspected PH in other patient categories remains unclear.

Our study has some limitations. First, ECG data before or after PEA was unavailable in a proportion of the CTEPH patients. While this missing data is likely random, the possibility of selection bias cannot be entirely ruled out. Second, there was a time gap between the ECGs and RHC, as it was not mandatory to conduct ECGs on the same day as the catheterization procedure. As a result in 10 patients, ECGs were conducted more than 90 days after right heart catheterization. However, we addressed this limitation by conducting a sensitivity analysis excluding these patients, which yielded similar results. Third, CMR data was only accessible in 20 patients. As a result, the sub-analysis involving CMR is underpowered, and definitive conclusions cannot be drawn. Lastly, (residual) PH was defined as mPAP ≥ 25 mmHg measured with RHC according to the current guideline at time of inclusion of this cohort [16]. Therefore applicability of our findings to CTEPH patients diagnosed using the 2022 definition of PH may be debatable. To address concerns regarding the applicability of the definition of residual PH, we conducted a sensitivity analysis classifying CTEPH patients with residual PH according to the 2022 definition, yielding similar results.

In conclusion, while we observed a correlation between VG-RVPO and increased pulmonary artery pressure in CTEPH patients before PEA, this correlation appears to diminish after PEA. The remodeling of the heart after PEA such as persistent abnormality in the composition of the heart or persistent RV hypertrophy despite normalization of the pulmonary artery pressure seems to clarify why our study did not demonstrate a relevant diagnostic value of VG-RVPO for detecting PH in CTEPH patients post-PEA. These findings suggest that the utility of VG-RVPO is limited in this context, highlighting the need for further research to explore alternative approaches to improve (non-invasive) follow-up of CTEPH patients post PEA.

## Supporting information

S1 Fig VG-RVPO and indexed RV mass pre- and post-PEA. Dashed line presents the threshold for normal VG-RVPO or indexed RV mass. Below the black dashed line are the normal values, above abnormal. Abbreviations: PEA, pulmonary endarterectomy; RV, right ventricle; VG-RVPO, ventricular gradient optimized for right ventricular pressure overload.
(TIF)

S1 Table Diagnostic accuracy of specific cut-off values, sensitivity analysis according to normal or abnormal RV mass. Abbreviations: PEA, pulmonary endarterectomy; PH, pulmonary hypertension; RV right ventricle; SD, standard deviation; VG-RVPO, ventricular gradient optimized for right ventricular pressure overload.
(DOCX)

S2 Table Overall accuracy of VG-RVPO; sensitivity analysis residual PH according to ESC 2022 PH guidelines *  1 patient excluded because of missing PVR data. Abbreviations: PEA, pulmonary endarterectomy; PH, pulmonary hypertension; SD, standard deviation; VG-RVPO, ventricular gradient optimized for right ventricular pressure overload.
(DOCX)

S3 Table Diagnostic accuracy of specific cut-off values; sensitivity analysis residual PH according to ESC 2022 PH guidelines. Abbreviations: PEA, pulmonary endarterectomy; PH, pulmonary hypertension; SD, standard deviation; VG-RVPO, ventricular gradient optimized for right ventricular pressure overload.
(DOCX)

S4 Table AUC ROC curve; sensitivity analysis residual PH according to ESC 2022 PH guidelines. Abbreviations: AUC, area under the curve; VG-RVPO, ventricular gradient optimized for right ventricular pressure overload.
(DOCX)

S5 Table Overall accuracy of VG-RVPO; sensitivity analysis ECG > 90 days after RHC excluded. Abbreviations: PEA, pulmonary endarterectomy; PH, pulmonary hypertension; SD, standard deviation; VG-RVPO, ventricular gradient optimized for right ventricular pressure overload.
(DOCX)

S6 Table Diagnostic accuracy of specific cut-off values; sensitivity analysis ECG > 90 days after RHC excluded. Abbreviations: PEA, pulmonary endarterectomy; PH, pulmonary hypertension; SD, standard deviation; VG-RVPO, ventricular gradient optimized for right ventricular pressure overload.
(DOCX)

S7 Table AUC ROC curve; sensitivity analysis ECGs > 90 days after RHC excluded. Abbreviations: AUC, area under the curve; VG-RVPO, ventricular gradient optimized for right ventricular pressure overload.
(DOCX)

S1 File Data availability. (CSV)

## Abbreviation

ΔVG-RVPO: Change in Ventricular Gradient Optimized for Right Ventricular Pressure Overload
ECG: Electrocardiogram
ESC: European Society of Cardiology
IQR: Interquartile Range
LEADS: Leiden ECG Analysis and Decomposition Software
mPAP: Mean Pulmonary Artery Pressure
MRI: Magnetic Resonance Imaging
NYHA: New York Heart Association
PAH: Pulmonary Arterial Hypertension
PEA: Pulmonary Endarterectomy
PH: Pulmonary Hypertension
PVR: Pulmonary Vascular Resistance
RHC: Right Heart Catheterization
RV: Right Ventricle
RVPO: Right Ventricular Pressure Overload
SD: Standard Deviation
VG: Ventricular Gradient
VG-RVPO: Ventricular Gradient Optimized for Right Ventricular Pressure Overload.
