# Supplementary figures and images for: The value of vector ECG in predicting residual pulmonary hypertension in CTEPH patients after pulmonary endarterectomy
Source: PLoS One. 2025 Feb 26;20(2):e0317826. doi: 10.1371/journal.pone.0317826 (PMC11864536; doi:10.1371/journal.pone.0317826)

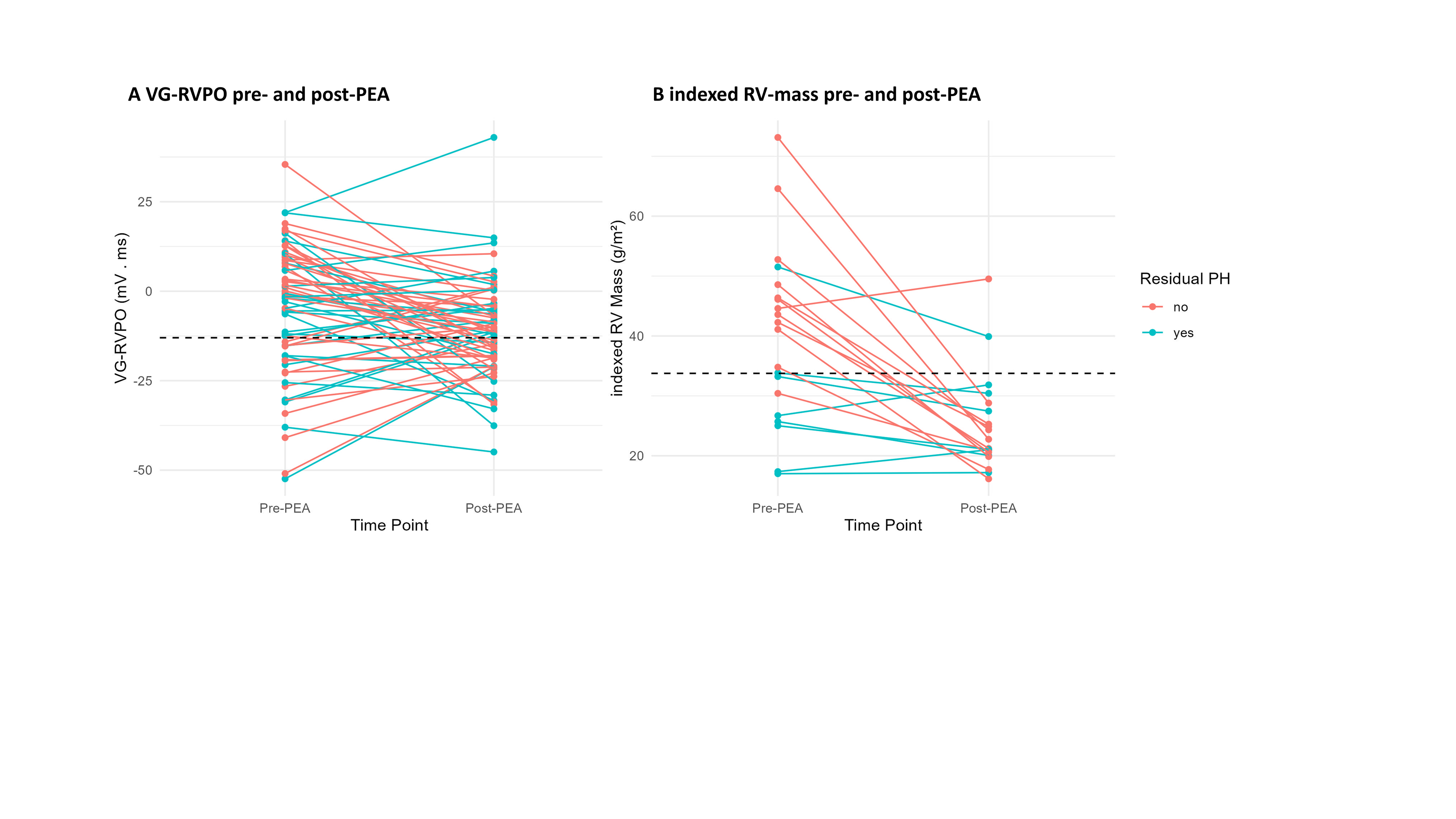

Supplement: S1 Fig — Dashed line presents the threshold for normal VG-RVPO or indexed RV mass. Below the black dashed line are the normal values, above abnormal. Abbreviations: PEA, pulmonary endarterectomy; RV, right ventricle; VG-RVPO, ventricular gradient optimized for right ventricular pressure overload. (TIF) [file pone.0317826.s001.tif]
