# Supplementary material for: The value of vector ECG in predicting residual pulmonary hypertension in CTEPH patients after pulmonary endarterectomy
Source: PLoS One. 2025 Feb 26;20(2):e0317826. doi: 10.1371/journal.pone.0317826 (PMC11864536; doi:10.1371/journal.pone.0317826)
Supplement: S1 Table — Abbreviations: PEA, pulmonary endarterectomy; PH, pulmonary hypertension; RV right ventricle; SD, standard deviation; VG-RVPO, ventricular gradient optimized for right ventricular pressure overload. (DOCX) [file pone.0317826.s002.docx]

**S1 Table. Diagnostic accuracy of specific cut-off values, sensitivity analysis according to normal or abnormal RV mass.**

| Cut-off value | | Normal RV mass (n=18) | | Abnormal RV mass (n=2) | |
| --- | --- | --- | --- | --- | --- |
|  |  | Patients without residual PH after PEA (n=11) | Patients with residual PH after PEA (n=7) | Patients without residual PH after PEA (n=1) | Patients with residual PH after PEA (n=1) |
| Abnormal follow-up VG-RVPO of ≥-13 mV·ms (previously defined cut-off value) | VG-RVPO normal, n (%) | 6 (54.55) | 3 (42.86) | 1 (100) | 0 (0) |
|  | VG-RVPO abnormal, n (%) | 5 (45.45) | 4 (57.14) | 0 (0) | 1 (100) |
| Abnormal follow-up VG-RVPO of ≥-14.7 mV·ms | VG-RVPO normal, n (%) | 6 (54.55) | 2 (28.57) | 1 (100) | 0 (0) |
|  | VG-RVPO abnormal, n (%) | 5 (45.45) | 5 (71.43) | 0 (0) | 1 (100) |
| Abnormal Δ VG-RVPO of ≥-24.9 mV·ms | VG-RVPO normal, n (%) | 4 (36.36) | 0 (0) | 0 (0) | 0 (0) |
|  | VG-RVPO abnormal, n (%) | 7 (63.64) | 7 (100) | 1 (100) | 1 (100) |

Abbreviations: PEA, pulmonary endarterectomy; PH, pulmonary hypertension; RV right ventricle; SD, standard deviation; VG-RVPO, ventricular gradient optimized for right ventricular pressure overload.
