# Supplementary material for: The value of vector ECG in predicting residual pulmonary hypertension in CTEPH patients after pulmonary endarterectomy
Source: PLoS One. 2025 Feb 26;20(2):e0317826. doi: 10.1371/journal.pone.0317826 (PMC11864536; doi:10.1371/journal.pone.0317826)
Supplement: S2 Table — Abbreviations: PEA, pulmonary endarterectomy; PH, pulmonary hypertension; SD, standard deviation; VG-RVPO, ventricular gradient optimized for right ventricular pressure overload. (DOCX) [file pone.0317826.s003.docx]

**S2 Table. Overall accuracy of VG-RVPO; sensitivity analysis residual PH according to ESC 2022 PH guidelines**

|  | All patients (n=65)* | Patients without residual PH after PEA (n=35) | Patients with residual PH after PEA (n=30) | Mean difference (95%CI) |
| --- | --- | --- | --- | --- |
| VG-RVPO at baseline (mV·ms), mean +- SD | -5.44 (18.2) | -0.26 (13.05) | -11.48 (21.4) | -11.2 (95% CI -20.2--2.24) |
| VG-RVPO during follow-up (mV·ms), mean +- SD | -10.9 (13.5) | -12.25 (10.42) | -9.32 (16.4) | 2.93 (95% CI -4.02-9.88) |
| Δ VG-RVPO (between baseline and during follow up) (mV·ms), mean +- SD | -5.46 (17.2) | -11.99 (14.2) | 2.16 (17.6) | 14.2 (95% CI 6.15-22.2) |

* 1 patient excluded because of missing PVR data Abbreviations: PEA, pulmonary endarterectomy; PH, pulmonary hypertension; SD, standard deviation; VG-RVPO, ventricular gradient optimized for right ventricular pressure overload.
