# Supplementary material for: The value of vector ECG in predicting residual pulmonary hypertension in CTEPH patients after pulmonary endarterectomy
Source: PLoS One. 2025 Feb 26;20(2):e0317826. doi: 10.1371/journal.pone.0317826 (PMC11864536; doi:10.1371/journal.pone.0317826)
Supplement: S3 Table — Abbreviations: PEA, pulmonary endarterectomy; PH, pulmonary hypertension; SD, standard deviation; VG-RVPO, ventricular gradient optimized for right ventricular pressure overload. (DOCX) [file pone.0317826.s004.docx]

**S3 Table. Diagnostic accuracy of specific cut-off values; sensitivity analysis residual PH according to ESC 2022 PH guidelines**

|  | | Patients without residual PH after PEA (n= 35) | Patients with residual PH after PEA (n=30) |
| --- | --- | --- | --- |
| Abnormal follow-up VG-RVPO of ≥-13 mV·ms (previously defined cut-off value) | VG-RVPO normal, n (%) | 18 (51.4) | 10 (33.3) |
|  | VG-RVPO abnormal, n (%) | 17 (48.6) | 20 (66.7) |
| Abnormal follow-up VG-RVPO of ≥-14.7 mV·ms | VG-RVPO normal, n (%) | 17 (48.6) | 9 (30) |
|  | VG-RVPO abnormal, n (%) | 18 (51.4) | 21 (70) |
| Abnormal Δ VG-RVPO of ≥-24.9 mV·ms | VG-RVPO normal, n (%) | 7 (20) | 2 (6.7) |
|  | VG-RVPO abnormal, n (%) | 28 (80) | 28 (93.3) |

Abbreviations: PEA, pulmonary endarterectomy; PH, pulmonary hypertension; SD, standard deviation; VG-RVPO, ventricular gradient optimized for right ventricular pressure overload.
