# Supplementary material for: The value of vector ECG in predicting residual pulmonary hypertension in CTEPH patients after pulmonary endarterectomy
Source: PLoS One. 2025 Feb 26;20(2):e0317826. doi: 10.1371/journal.pone.0317826 (PMC11864536; doi:10.1371/journal.pone.0317826)
Supplement: S4 Table — Abbreviations: AUC, area under the curve; VG-RVPO, ventricular gradient optimized for right ventricular pressure overload. (DOCX) [file pone.0317826.s005.docx]

**S4 Table. AUC ROC curve; sensitivity analysis residual PH according to ESC 2022 PH guidelines.**

|  | AUC (95%CI |
| --- | --- |
| follow-up VG-RPVO | 0.561 (0.416-0.561) |
| follow-up VG-RVPO ≥-13 mV·ms | 0.590 (0.47-0.59) |
| follow-up VG-RVPO ≥-14.7 mV·ms | 0.593 (0.474-0.711) |
| Δ VG-RVPO | 0.774 (0.656-0.774) |
| Δ VG-RVPO ≥-24.9 mV·ms | 0.567 (0.486-0.711) |

Abbreviations: AUC, area under the curve; VG-RVPO, ventricular gradient optimized for right ventricular pressure overload.
