# Supplementary material for: The value of vector ECG in predicting residual pulmonary hypertension in CTEPH patients after pulmonary endarterectomy
Source: PLoS One. 2025 Feb 26;20(2):e0317826. doi: 10.1371/journal.pone.0317826 (PMC11864536; doi:10.1371/journal.pone.0317826)
Supplement: S5 Table — Abbreviations: PEA, pulmonary endarterectomy; PH, pulmonary hypertension; SD, standard deviation; VG-RVPO, ventricular gradient optimized for right ventricular pressure overload. (DOCX) [file pone.0317826.s006.docx]

**S5 Table. Overall accuracy of VG-RVPO; sensitivity analysis ECG >90 days after RHC excluded.**

|  | All patients (n=56) | Patients without residual PH after PEA (n=32) | Patients with residual PH after PEA (n=24) | Mean difference (95%CI) |
| --- | --- | --- | --- | --- |
| VG-RVPO at baseline (mV·ms), mean +- SD | -5.91 (17.8) | -3.55 (18.37) | -9.04 (16.9) | -5.48 (95% CI -15.0-4.02) |
| VG-RVPO during follow-up (mV·ms), mean +- SD | -11.18 (14.1) | -11.64 (10.1) | -10.57 (18.4) | 1.06 (95% CI -7.26-9.39) |
| Δ VG-RVPO (between baseline and during follow up) (mV·ms), mean +- SD | -5.28 (17.5) | -8.08 (18.1) | -1.53 (16.2) | 6.55 (95% CI -2.68-15.8) |

Abbreviations: PEA, pulmonary endarterectomy; PH, pulmonary hypertension; SD, standard deviation; VG-RVPO, ventricular gradient optimized for right ventricular pressure overload.
