# Supplementary material for: The value of vector ECG in predicting residual pulmonary hypertension in CTEPH patients after pulmonary endarterectomy
Source: PLoS One. 2025 Feb 26;20(2):e0317826. doi: 10.1371/journal.pone.0317826 (PMC11864536; doi:10.1371/journal.pone.0317826)
Supplement: S6 Table — Abbreviations: PEA, pulmonary endarterectomy; PH, pulmonary hypertension; SD, standard deviation; VG-RVPO, ventricular gradient optimized for right ventricular pressure overload. (DOCX) [file pone.0317826.s007.docx]

**S6 Table. Diagnostic accuracy of specific cut-off values; sensitivity analysis ECG >90 days after RHC excluded.**

|  | | Patients without residual PH after PEA (n= 32) | Patients with residual PH after PEA (n=24) |
| --- | --- | --- | --- |
| Abnormal follow-up VG-RVPO of ≥-13 mV·ms (previously defined cut-off value) | VG-RVPO normal, n (%) | 19 (50) | 10 (35.7) |
|  | VG-RVPO abnormal, n (%) | 19 (50) | 18 (64.3) |
| Abnormal follow-up VG-RVPO of ≥-14.7 mV·ms | VG-RVPO normal, n (%) | 15 (46.9) | 8 (33.3) |
|  | VG-RVPO abnormal, n (%) | 17 (53.1) | 16 (66.7) |
| Abnormal Δ VG-RVPO of ≥-24.9 mV·ms | VG-RVPO normal, n (%) | 6 (18.8) | 1 (4.17) |
|  | VG-RVPO abnormal, n (%) | 26 (81.3) | 23 (95.8) |

Abbreviations: PEA, pulmonary endarterectomy; PH, pulmonary hypertension; SD, standard deviation; VG-RVPO, ventricular gradient optimized for right ventricular pressure overload.
