# Supplementary material for: The value of vector ECG in predicting residual pulmonary hypertension in CTEPH patients after pulmonary endarterectomy
Source: PLoS One. 2025 Feb 26;20(2):e0317826. doi: 10.1371/journal.pone.0317826 (PMC11864536; doi:10.1371/journal.pone.0317826)
Supplement: S7 Table — Abbreviations: AUC, area under the curve; VG-RVPO, ventricular gradient optimized for right ventricular pressure overload. (DOCX) [file pone.0317826.s008.docx]

**S7 Table. AUC ROC curve; sensitivity analysis ECGs >90 days after RHC excluded.**

|  | AUC (95%CI |
| --- | --- |
| follow-up VG-RPVO | 0.527 (0.365-0.527) |
| follow-up VG-RVPO ≥-13 mV·ms | 0.562 (0.43-0.562) |
| follow-up VG-RVPO ≥-14.7 mV·ms | 0.568 (0.437-0.698) |
| Δ VG-RVPO | 0.628 (0.478-0.628) |
| Δ VG-RVPO ≥-24.9 mV·ms | 0.573 (0.493-0.698) |

Abbreviations: AUC, area under the curve; VG-RVPO, ventricular gradient optimized for right ventricular pressure overload.
